# The long-term effects of hyaluronic acid on hemiplegic shoulder pain and injury in stroke patients: A randomized controlled study

**DOI:** 10.1097/MD.0000000000012078

**Published:** 2018-08-21

**Authors:** Yu-Chi Huang, Chau-Peng Leong, Hui-Hsin Tso, Mei-Ju Chen, Mei-Yun Liaw, Han-Chin Hsieh, Lin-Yi Wang, Chia-Hao Hsu

**Affiliations:** Department of Physical Medicine and Rehabilitation, Kaohsiung Chang Gung Memorial Hospital and Chang Gung University College of Medicine, Kaohsiung, Taiwan.

**Keywords:** hemiplegic shoulder pain, hyaluronic acid, sonography, stroke

## Abstract

**Background:**

Hemiplegic shoulder pain (HSP) is one common complication after stroke. The interferes with the functionality of the affected shoulder in patients with stroke during rehabilitation. Hyaluronic acid (HA) could have positive effects on pain relief and shoulder motion in stroke patients with hemiplegic shoulders. We investigated long-term benefits of HA injection in stroke patients with HSP and rotator cuff injury.

**Methods:**

A randomized, double-blinded controlled trial was conducted in one medical center. The stroke patients with HSP and rotator cuff injury were randomized and allocated to the control (n = 9) and experimental (n = 18) groups. The control and the experimental groups received ultrasound-guided subacromial 0.9% sodium chloride and HA injections, respectively. All injections were performed once per week for 3 weeks. The associated upper extremity functional assessments, shoulder pain scale, and sonography findings on affected shoulders were measured before interventions and at the 4th and 12th week after local injections.

**Results:**

The visual analog scale (VAS) scores of HSP were significantly reduced in the control and experimental groups at the 4th week following intervention. Additionally, the VAS score at the 12th week was also significantly reduced in the experimental group. Significant differences were found in the hyperemia occurrence in the subscapularis tendon at the 12th week after intervention (*P* = .018) and in the severity of hyperemia in the long head of the biceps tendon (*P* = .042) and the subscapularis tendon after intervention (*P* = .014).

**Conclusion:**

Subacromial HA injections might provide longer HSP reduction and decrease in hyperemia reactions at the long head of biceps tendon and subscapularis tendon in stroke patients with HSP and tendon injury.

## 1 Introduction

Hemiplegic shoulder pain (HSP) is a common comorbidity in stroke patients with hemiplegia. HSP impedes functional outcomes due to its negative effects on motor function of affected shoulders, daily activities, and length of hospital stay after stroke.^[1–3]^ The pathogenesis of HSP is still not well determined and previous investigators proposed that impaired shoulder function, restricted shoulder motion, shoulder subluxation, shoulder spasticity, reflex sympathetic dystrophy, and rotator cuff injuries are contributors of HSP.^[4–6]^ There are some other interventions available, including medications, local injections, rehabilitation, taping, and exercises, to treat HSP in patients with stroke.^[7–15]^ Rotator cuff injury is considered to be one important factor related to HSP in patients with subacute stroke.^[5,16]^ Clinically, the patients with shoulder pain may receive steroid or hyaluronic acid (HA) injections to alleviate pain associated with local inflammation.^[9,12,17–19]^ The reduction of HSP after steroid injections was significantly effective, whereas the long-term adverse effects of steroid injections, including skin depigmentation, tissue degeneration, and tendon rupture, were also reported.^[20]^ Since stroke patients with HSP were frequently associated with rotator cuff injury, we considered HA injection could be an alternative and safer intervention for reduction of pain and inflammation. Moreover, HA protects the cartilage and inhibits its degeneration and improves metabolism in the synovial fluid, tendons, and ligaments.^[21–25]^ Intraarticular HA injection in patients with shoulder rotator cuff disorder, such as supraspinatus tendinitis, resulted in positive effects on shoulder pain and motion and activities of daily living.^[26]^ Regarding patients with rotator cuff injury, some researchers found that subacromial HA injection resulted in positive effect on pain reduction lasting from 6 to 12 weeks.^[18,27]^ According to our pilot study, we found that HA injection led to the short-term benefits on HSP and motor function recovery in stroke patients with HSP and rotator cuff injury.^[28]^ There was no further study to explore the long-term effects of HA injection on HSP and shoulder injury in stroke patients with hemiplegia. The aim of this study was to investigate the long-term effects of HA injection for reducing pain and inflammation reactions of rotator cuff and biceps tendon at the 12th week of the study in subacute stroke patients with hemiplegic shoulder injury and pain.

## 2 Materials and methods

### 2.1 Study design

The study was a double-blinded, randomized controlled trial (clinical trial registration number NCT02465853). A parallel-group design with allocation concealment was used in this study. It was originally designed that the ratio of the enrolments in experimental group to control group was 2 to 1. Before the beginning of this trial, a serial number had been set up and was randomly categorized into either experimental group or control group by the research assistant. Therefore, the patients would be categorized into one of the group based on the number they picked. The participants and their caregivers would not receive any information related to the medicine they injected. Therapists who are responsible for assessing outcomes were not involving during the intervention process. After the trial commencement, there was no significant change being made. All the participants were enrolled between February 2015 and January 2017 at a rehabilitation unit of a single medical center in Taiwan.

### 2.2 Participants

**Figure 1:**
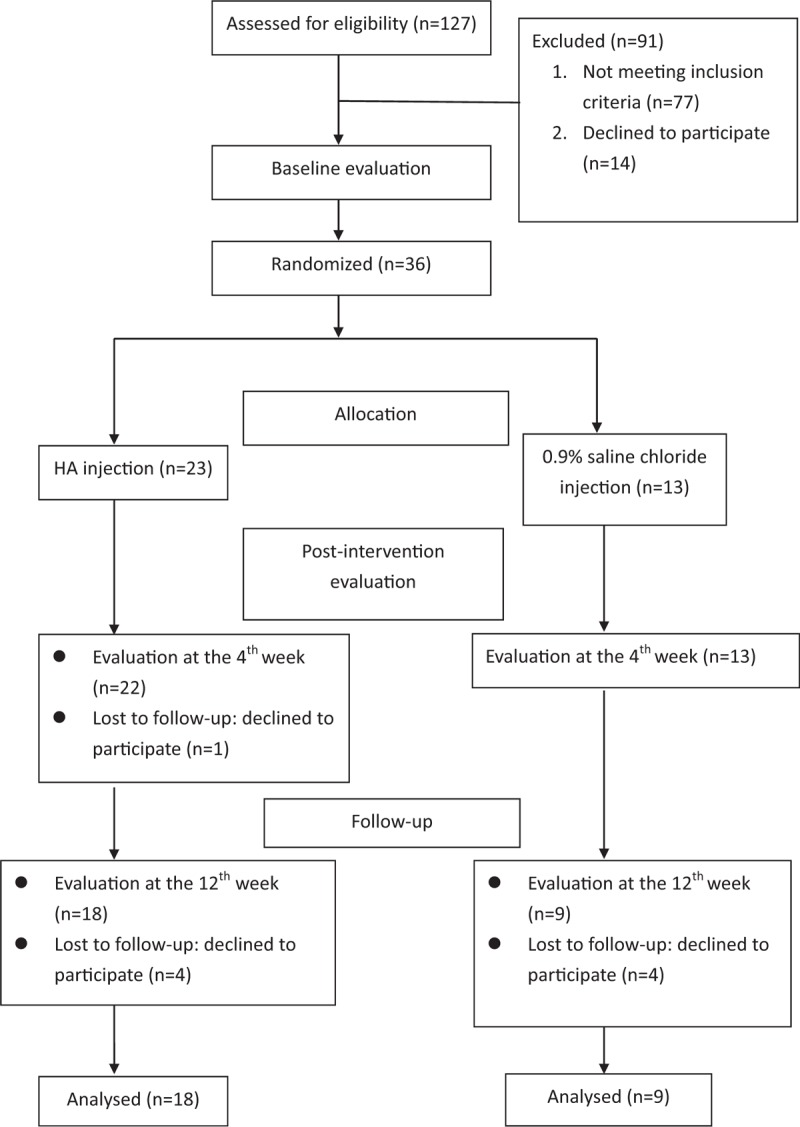
Flow diagram of eligible patients selected for this study.

Of the 127 stroke patients with HSP who admitted or those who visited the rehabilitation unit at a medical center, 27 stroke patients met the inclusive criteria and were recruited and completed all the procedures in this study (Fig. 1). The inclusion criteria are listed below: stroke patients with unilateral hemiplegia (stroke onset within 6 months), presence of HSP assessed by the visual analog scale (VAS), the shoulder pain scale score equal to or more than 3, and weakened shoulders with soft tissue injuries revealed by musculoskeletal sonography. The exclusion criteria were taking anticoagulation therapy, shoulder pain or soft tissue injury before stroke, other neuromuscular disease, or impaired communication that does not allow understanding or cooperating within the procedures. This clinical trial was approved by the Institutional Review Board at our hospital. All investigators followed the ethical principles for medical research according to the Declaration of Helsinki 2013. The patients understood the study protocol and procedures, and the written informed consent was obtained from each participant before trial execution.

### 2.3 Interventions

We randomly allocated the stroke patients into the control and experimental groups before interventions. A research assistant used sequentially numbered and sealed envelopes to perform randomization. In the control group, 9 patients received a 2.5 mL 0.9% sodium chloride injection into the subdeltoid bursa under ultrasound guidance. In the experimental group, 18 patients with stroke received a 2.5 mL sodium hyaluronate injection (ARTZ Dispo, Seikagaku, Tokyo, Japan) into the subdeltoid bursa under ultrasound guidance. Total 3 injections for each patient were performed (once a week for 3 weeks) and administered by 1 experienced physiatrist. The participants were blinded to the content of local injections in this study.

### 2.4 Outcome measures

A physical therapist, also blinded to the content of local injections, assessed the related parameters before, 4- and 12-week after treatment. These included shoulder spasticity, measured by the modified Ashworth scale^[29,30]^; shoulder subluxation, examined by a fingerbreadth to evaluate the distance between the lateral border of the acromion and the proximal part of humeral head; pain-free range of motion of the hemiplegic shoulder, measured with a goniometer; Fugl-Meyer assessment for the upper extremity (FMA-UE)^[31]^; and the severity of HSP. The medications for pain control and rehabilitation sessions were recorded during the 12-week period in this study. The physical and occupational therapists were informed about injured shoulders in the participants and also had been instructed proper techniques during performing exercises. The presence of HSP was noted while resting or during passive shoulder motion, and the pain severity was assessed by a 10-cm VAS. All shoulder sonographic images were evaluated by the same experience physiatrist certificated by Taiwan Society of Ultrasound in Medicine. The physician performed a musculoskeletal sonography using a 9 to 14 MHz linear-array transducer (ACUSON S2000; Siemens, Malvern, PA). The long head of the biceps tendon, subscapularis tendon, supraspinatus, and infraspinatus tendons, and subdeltoid bursa were investigated in this study. Their echogenicity and the thickness of each tendon, the fluids surrounding tendons, or in the bursa, were evaluated and measured. We assessed inflammatory responses of these soft tissues in hemiplegic shoulders by color Doppler sonography, and recorded the hyperemia level according to the following grading: the color Doppler sonography signal was scored as grade 0 (no color flow signal), grade 1 (<3 color spots or a short line), grade 2 (3–6 color spots or short lines), or grade 3 (>6 color spots or color lines).^[32]^

The physical assessments, shoulder pain, and shoulder sonography were evaluated at baseline, the 4th and 12th week after intervention for each participant. After the trial commencement, there were no significant changes about outcome measure being made.

### 2.5 Statistical analysis

Categorical variables between groups were compared using Fisher exact test, and continuous variables were compared by Mann–Whitney *U* test. Variables before and after intervention within group were compared by the Wilcoxon signed-rank test. Within-group comparisons of the structural findings and hyperemia level on shoulder sonography were analyzed by generalized linear models and the Friedman test, respectively. A *P*-value less than .05 was set as statistically significant. All statistical tests were performed using SPSS 19.0 (SPSS, Inc., Chicago, IL).

## 3 Results

**Figure 2:**
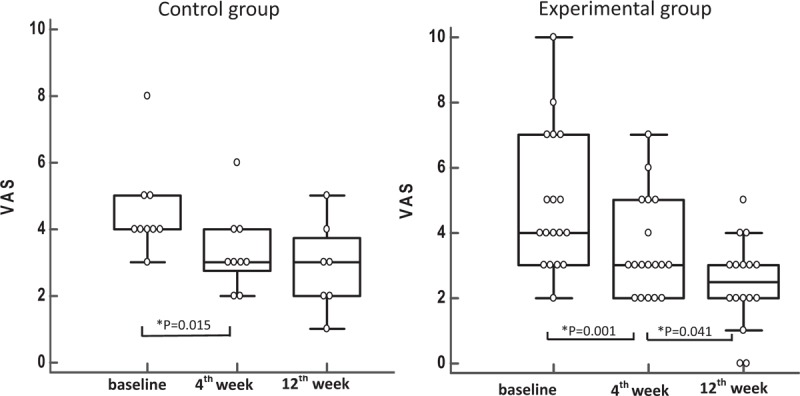
Comparing the VAS score from time to time within each group. The vertical axis represents VAS scores; the transverse axis represents the 3 scheduled times of the assessments. VAS = visual analog scale. ^∗^Represents significant improvement (*P* < .05).

The data was collected and analyzed in April 2017, when the participants completed the injections and following assessments after intervention. There were no reports of harmful side effects after receiving subacromial HA or normal saline injections in this trial. Table 1 shows the clinical characteristics, including demography, shoulder spasticity, subluxation, upper extremity function, shoulder motion, and pain levels before intervention in the participants and presents the medication and rehabilitation conditions in this study. There were no significant differences in clinical variables between the control and experimental groups before intervention. In the study, no significant differences were found in medication and rehabilitation program between these 2 groups. In within-group comparisons, no significant differences were found in shoulder spasticity, subluxation, shoulder flexion, and abduction at the 4th and 12th week after intervention in both control and experimental groups (Table 2). There were significant differences in VAS and FMA-UE scores between baseline and the 4th week in the control group (*P* = .015 and .041, respectively; Table 2; Fig. 2). In the experimental group, there were significant differences in VAS and FMA-UE scores between baseline and the 4th week (*P* = .001 and .009, respectively), and in VAS scores between the 4th and 12th week after intervention (*P* = .041; Table 2; Fig. 2).

**Table 1:**
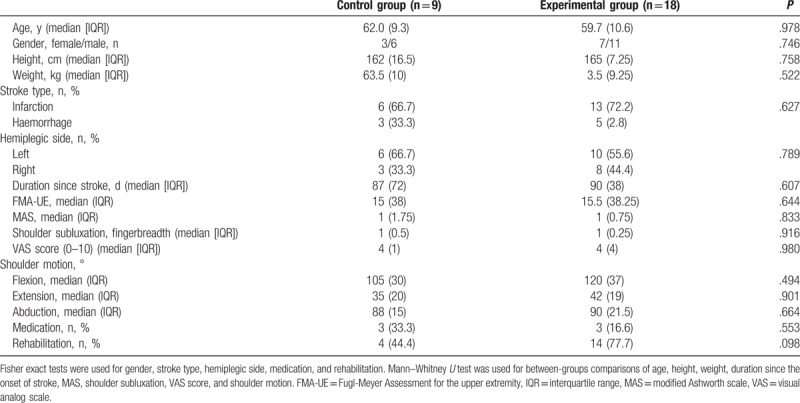
Clinical characteristics of stroke patients in the control and experimental groups.

**Table 2:**
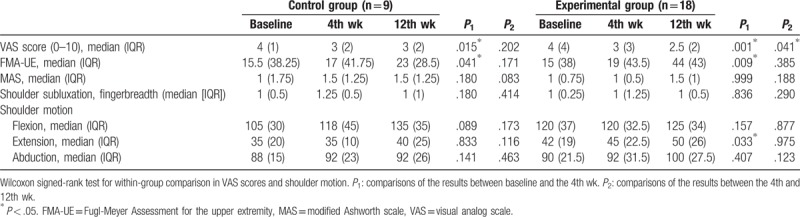
Comparisons of VAS scores and shoulder motion in the control and experimental groups.

Comparisons of shoulder structural changes on hemiplegic shoulders between baseline and after intervention in the control and experimental groups are presented in Tables 3 and 4. There were no significant differences in the incidence of tendon tear, tenosynovitis, and bursitis at the long head of biceps tendon and rotator cuff, but significant differences were observed in the incidence of the hyperemia in the subscapularis tendons on shoulder sonography between the 4th and 12th week after intervention in the experimental group (*P* = .018; Table 3). The levels of hyperemia at tendons were significantly different at the long head of the biceps tendon and the subscapularis tendon after intervention in the experimental group (*P* = .042 and .014, respectively; Table 4).

**Table 3:**
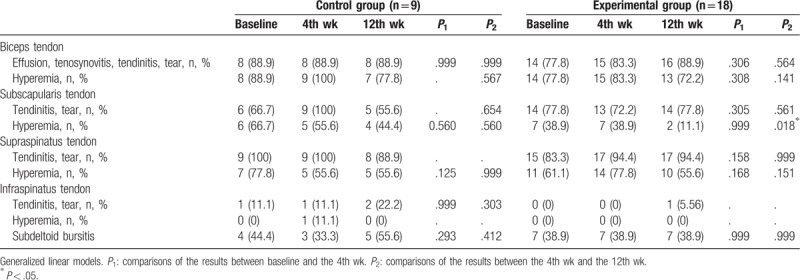
Comparison of the structural findings on shoulder sonography in the control and experimental groups.

**Table 4:**
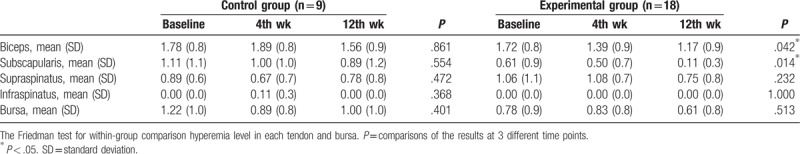
Comparison of hyperemia levels on shoulder sonography in the control and experimental groups.

## 4 Discussion

In this trial, subacromial HA injection was used to manage the HSP and injury in subacute stroke patients with hemiplegic shoulders. According to the findings of our previous pilot study, subacromial HA injection could provide some benefits on pain reduction and motor function improvement of hemiplegic shoulders.^[28]^ In this longitudinal study, the results supported that the HA injection could not only provide HSP reduction lasting for 12 weeks, but also decrease the prevalence of inflammatory reactions in the subscapularis tendon at the 12th week after intervention. Furthermore, we also found that the levels of inflammatory responses at the long head of the biceps tendon and the subscapularis tendon were progressively decreased in the following 12 weeks after HA injection. In a randomized control trial, Rah et al reported that subacromial corticosteroid injection could reduce HSP and improve shoulder motion which had been lasting for 8 weeks in chronic stroke patients.^[33]^ The efficacy and duration for pain reduction by subacromial corticosteroid injection was similar to the benefits for pain relief by subacromial HA injection in subacute stroke patients presented in our study.

Stroke patients with flaccid shoulders have a high tendency to get an injury of the weakened shoulders while performing daily activities and rehabilitation. Steroids could provide effective pain reduction due to antiinflammatory effects. However, adverse effects after local injections of steroids were reported, including paler skin, tissue atrophy, tendon rupture, and even cartilage damage.^[20,34–36]^ HA may play an antiinflammatory role by inhibiting proinflammatory cytokines and cyclooxygenase-2/prostaglandin E2 production in rotator cuff injury.^[18,37]^ We considered HA could decrease inflammation leading to pain reduction, and HA did not do further harm at the soft tissues and bony structures of hemiplegic shoulders in stroke patients with high risk of rotator cuff injury. Therefore, we used subacromial HA injection instead of steroid injection to resolve inflammatory responses and to treat HSP in stroke patients with hemiplegic shoulder injury and pain.

This is the first longitudinal study to explore long-term effect of pain reduction and also to present its antiinflammatory responses on injured tendons by shoulder sonography in stroke patients with HSP and injury. There were significant reductions in hyperemia level surrounding or in the long head of the biceps tendon and the subscapularis tendon. Higher prevalence and inflammatory reactions of the supraspinatus tendon injury on hemiplegic shoulders did not change after injections. The explanation for this finding is that the supraspinatus tendon is at a higher risk of injury during activities and exercises because it is a key muscle involved in shoulder exercises and rehabilitation. Besides, we also found a decline in pain score of HSP during a 3-month period after HA injections. Subacromial HA injections could provide a longer antiinflammatory effect for stroke patients with injured tendons. It means that pain-reduction process continued during the following 3 months after interventions, which may be associated with decreased hyperemia at some of hemiplegic shoulder tendons due to HA local antiinflammatory effect.

The limitations in this study are listed below. First, we only recruited subacute stroke patients from 1 medical center and the total number of participants was limited. Second, the details and durations of performing various exercises program on injured shoulders, which may lead to further hemiplegic shoulder injury, were not exactly recorded. Furthermore, a 6-month period or longer follow-up was not assessed in stroke patients with HSP and injury. In future, more stroke patients with HSP and injury from multiple medical centers should be considered to evaluate clinical benefits of subacromial HA injections.

## 5 Conclusions

In summary, subacromial HA injections might provide a practical and effective therapy for stroke patients with shoulder injury and pain to relief HSP associated with decreased hyperemia reactions. We suppose that 3 subacromial HA local injections could offer some benefits for a longer period.

## Acknowledgment

The authors thank the coworkers in Chang Gung Memorial Hospital

## Author contributions

**Conceptualization:** Yu-Chi Huang, Chau-Peng Leong, Mei-Yun Liaw, Lin-Yi Wang.

**Data curation:** Hui-Hsin Tso, Mei-Ju Chen, Han-Chin Hsieh, Chia-Hao Hsu.

**Formal analysis:** Hui-Hsin Tso.

**Investigation:** Yu-Chi Huang.

**Methodology:** Yu-Chi Huang, Chau-Peng Leong, Mei-Ju Chen, Mei-Yun Liaw, Han-Chin Hsieh, Lin-Yi Wang.

**Project administration:** Yu-Chi Huang.

**Supervision:** Chau-Peng Leong.

**Writing – original draft:** Yu-Chi Huang.

**Writing – review & editing:** Yu-Chi Huang, Chau-Peng Leong, Mei-Yun Liaw, Lin-Yi Wang, Chia-Hao Hsu.
